# Identifying priorities in knowledge translation from the perspective of trainees: results from an online survey

**DOI:** 10.1186/s13012-015-0282-5

**Published:** 2015-06-21

**Authors:** Kristine Newman, Dwayne Van Eerd, Byron J. Powell, Robin Urquhart, Evelyn Cornelissen, Vivian Chan, Shalini Lal

**Affiliations:** Daphne Cockwell School of Nursing, Faculty of Community Services, Ryerson University, Toronto, M5B 2K3 Canada; Institute for Work and Health, Toronto, Canada; School of Public Health and Health Systems, University of Waterloo, Waterloo, Canada; Department of Psychiatry, Perelman School of Medicine, University of Pennsylvania, Pennsylvania, USA; Department of Surgery, Dalhousie University, Halifax, Canada; Department of Family Practice, Faculty of Medicine, University of BC, Vancouver, BC Canada; Department of Medicine, Quality and Safety, Vancouver Coastal Health, Vancouver, Canada; School of Rehabilitation, Faculty of Medicine, University of Montreal, Montreal, Canada

**Keywords:** Knowledge translation, Trainee, Research priorities

## Abstract

**Background:**

The need to identify priorities to help shape future directions for research and practice increases as the knowledge translation (KT) field advances. Since many KT trainees are developing their research programs, understanding their concerns and KT research and practice priorities is important to supporting the development and advancement of KT as a field. Our purpose was to identify research and practice priorities in the KT field from the perspectives of KT researcher/practitioner trainees.

**Findings:**

Survey response rate was 62 % (44/71). Participants were mostly Canadian graduate students, post-doctoral fellows, residents, and learners from various disciplines; the majority was from Ontario (44 %) and Quebec (20 %). Seven percent (5/71) were from other countries including USA, UK, and Switzerland. Seven main KT priority themes were identified: determining the effectiveness of KT strategies, technology use, increased key stakeholder involvement, context, theory, expand *ways of inquiry*, and sustainability.

**Conclusions:**

Overall, the priorities identified by the trainees correspond with KT literature and with KT experts’ views. The trainees appeared to push the boundaries of current KT literature with respect to creative use of communication technologies research.

**Electronic supplementary material:**

The online version of this article (doi:10.1186/s13012-015-0282-5) contains supplementary material, which is available to authorized users.

## Findings

### Purpose and methods

The knowledge translation (KT) field has advanced rapidly in the last decade. Given that KT trainees are developing programs of research, understanding their priorities and concerns for KT research and practice is useful to advancing the KT field. This report defines KT as the “iterative, timely, and effective process of integrating best evidence into the routine practice of patients, practitioners, health-care teams, and systems” [1]. Indeed, working with their mentors, they may identify novel ways of viewing KT challenges and new approaches to solving them. The purpose of this study was to identify priorities for KT research and practice from the perspectives of KT researcher and practitioner trainees. Trainee perspectives can complement expert views expressed in the KT literature [2, 3].

A Web-based survey was used to identify KT trainees’ priorities related to KT practice and research. The 22 items included demographics, geographic location, career stage, KT experience, KT research priorities, and KT topics unworthy of further research/exploration. A purposive sample of trainees affiliated with the KT Trainee Collaborative (KTTC) [4] and 2013 KT Canada Summer Institute [2] was e-mailed a survey link through FluidSurveys™. Non-responders were sent three reminder e-mails 7, 14, and 21 days after the initial e-mail [5]. Responses to open-ended questions were analyzed using qualitative content analysis [6, 7]. The open-ended responses were reviewed and coded independently by two investigators [BJP, VC] who revised codes as general categories emerged. The codes were discussed and disagreements were reviewed in detail by coders until agreement was reached. The codes were then grouped thematically. The study team reviewed, discussed, and refined the themes to ensure they accurately represented the participants’ responses.

The Hamilton Integrated Research Ethics Board approved the study procedures.

## Results

We obtained a response rate of 62 % (44/77), and the respondents completed 91 % of the questions. The respondents self-identified as Masters or PhD students (60 %) or faculty (14 %), with the remainder (26 %) identifying as practitioners, clinician scientists, and fellows. Many respondents reported being involved in both KT research and practice (45 %) or solely KT research (41 %), with the remainder involved in KT practice or other (14 %).

The data were categorized into seven main themes related to KT priorities. Many of these priorities relate to one another, as depicted in Additional file 1.

### Determining the effectiveness of KT strategies

The most frequently mentioned KT priority concerned developing generalizable knowledge about the effectiveness of KT strategies in various contexts. The respondents spoke to the need for more empirical testing of KT strategies (“I believe that there needs to be a greater number of good quality, theory-based KT interventional studies conducted to improve our understanding of uptake and spread of knowledge”) [respondent D]. Many suggested testing whether tailored KT strategies are more effective than generic, one-size-fits-all strategies. The respondents emphasized the importance of adapting existing strategies from behavior change research (“many people do not benefit from this [the theoretical and empirical work related to behavior change] wisdom.”) [respondent N].

### Use of technology

The respondents frequently reported the need to capitalize on technologies such as the Web, mobile phone applications, health informatics, and social media in KT research and practice. One respondent wrote: “KT is about communication and the new technologies provide opportunities to test and understand the dilemma of the knowledge to practice gap differently” [respondent E]. The respondents highlighted the potential benefits and importance of sharing data through technology and online: “Online sharing is very easy, but the ‘open source’ mentality is far from the norm in … health research. On a positive note, KT researchers are very open in general, since they value collaboration and interdisciplinarity” [respondent N].

### Increased involvement of key stakeholders

The respondents noted the importance of more stakeholder involvement in various aspects of KT. This included developing partnerships with key stakeholders, such as commissioning bodies and a broad range of end users (e.g., policy makers, health-care providers, and patients) as well as across countries. One respondent stated, “it is not always clear either to practitioners that their particular local/lay knowledge is always welcome in academic research” [respondent AA]. Similarly, as another stated, “we often forget our main partner, the patient…there is a world that we need to explore there” [respondent K]. Lastly, the respondents acknowledged that stakeholder participation is limited by academic culture, which does not reward academic researchers for participating in KT practice. As one respondent [F] stated:

Academia creates incentives for publication, which we know is not an adequate strategy for effective KT. Creating solid partnerships takes a lot of time and resources, but those efforts are not valued and recognized in academia.

### Importance of context

The respondents prioritized assessing contextual elements in two ways. First, the respondents suggested that research is needed to clarify contextual constructs and develop methods for collecting, analyzing, and acting on data about contexts. For instance, the respondents prioritized pre-implementation assessments, implementation barriers, and feasibility of implementation. Second, many respondents suggested that specific contexts (or settings) require more KT research, such as dementia care, primary care, nutrition, chronic care, health-care organizations, rural settings, and low-resourced settings. For example, one respondent [F] challenged the KT research community to pay more attention to the needs of Indigenous peoples:

I believe the CIHR model for KT does not go far enough in recognizing and valuing different cultural (and other) factors that are necessary for effective KT with our Indigenous people—a true ethical problem in a time when health problems are dire for many Indigenous communities.

Finally, the respondents recommended that more attention be given to how evidence can be adapted to local contexts.

### Importance of theory

Several respondents’ priorities related to the use of theory in KT research and practice, stating that using theory is a prerequisite for quality KT research. They expressed their belief that using theories from diverse disciplines and perspectives is important and also suggested using “theoretical frameworks to identify the ‘key components’ or drivers in knowledge exchange” [respondent AJ]. While some respondents suggested specific theories (e.g., complexity theory), others referred more generally to the importance of integrating theory and practice.

Sixteen of the 44 respondents responded that there needs to be less emphasis on developing *new* KT frameworks/models and more emphasis on testing, refining, and improving those that already exist.

### Expand our *ways of inquiry*

Many respondents identified research priorities related to expanding our *ways of inquiry*. One respondent felt “we need to explore other modes of inquiry that are more finely attuned to the particular that shed light on specific relationships between actual people” [respondent X]. In addition, many suggested embracing a broader array of research methodologies and approaches underutilized in KT research, such as social network analysis, economic evaluation, mixed methods, and qualitative research. As one respondent [AL] noted,

KT is predominantly supported from a more traditional research perspective yet continuously, research indicates that the most critical component to the success or failure of a KT project or strategy is contextual. Qualitative research can more effectively get at context.

The respondents also prioritized development of valid and reliable outcome measures, including those for contextual elements (e.g., organizational readiness for change), complex interventions, service-system outcomes (e.g., timeliness), implementation outcomes (e.g., sustainability), and downstream effects of KT efforts on end users and health-care teams. They also expressed the importance of using evaluation frameworks and of routinely conducting evaluations of KT efforts.

Finally, the respondents prioritized improved descriptions of KT processes and research through development of reporting guidelines specific to KT. Reporting guidelines were suggested in response to a perceived problem in the literature: “limitations of syntheses are often related to intervention reporting” [respondent M].

### Sustainability

The respondents indicated that sustainability is a top priority at the design phase of any KT research or practice initiative. Many mentioned measuring sustainability of KT efforts as a priority. One respondent acknowledged, “We don't know if it is our efforts are sustainable, or even if they should be” [respondent K]. Other respondents noted the need for the development and testing of specific KT strategies to enhance sustainability.

## Conclusions

Overall, KT trainees identified KT research and practice priorities that align closely with those noted by KT experts in the KT literature. These include understanding context and contextual factors [8, 9], using theory in research and practice [10–13], evaluating effectiveness of KT strategies [3, 14–17], considering factors related to sustainability [18, 19], and employing new approaches to evaluation or *ways of inquiry* to better understand KT [3, 20–25]. Specifically, Eccles et al. [3] noted that identification, development, refinement, and testing of KT strategies have been prioritized by federal governments and there have been calls to utilize mixed methods and qualitative approaches to understand the nuances of contexts and processes related to KT. We determined trainee priorities through an empirical process to complement the views of experienced KT experts. These priorities reinforce the need to move KT research and practice forward in a number of strategic areas. These priorities will certainly not be the last word. Attending to these areas creatively will undoubtedly lead to the identification of further priorities and, in the process, help to strengthen the science and practice of KT.

## Additional file

Additional file 1:
**Identified KT Priorities.** Categorized seven main themes related to KT priorities

## Abbreviations

KT: knowledge translation
KTSI: 2013 KT Canada Summer Institute
KTTC: Knowledge Translation Trainee Collaborative
