# Supplementary figures and images for: Identifying priorities in knowledge translation from the perspective of trainees: results from an online survey
Source: Implement Sci. 2015 Jun 21;10:92. doi: 10.1186/s13012-015-0282-5 (PMC4475286; doi:10.1186/s13012-015-0282-5)

Identified  
KT  
Priorities

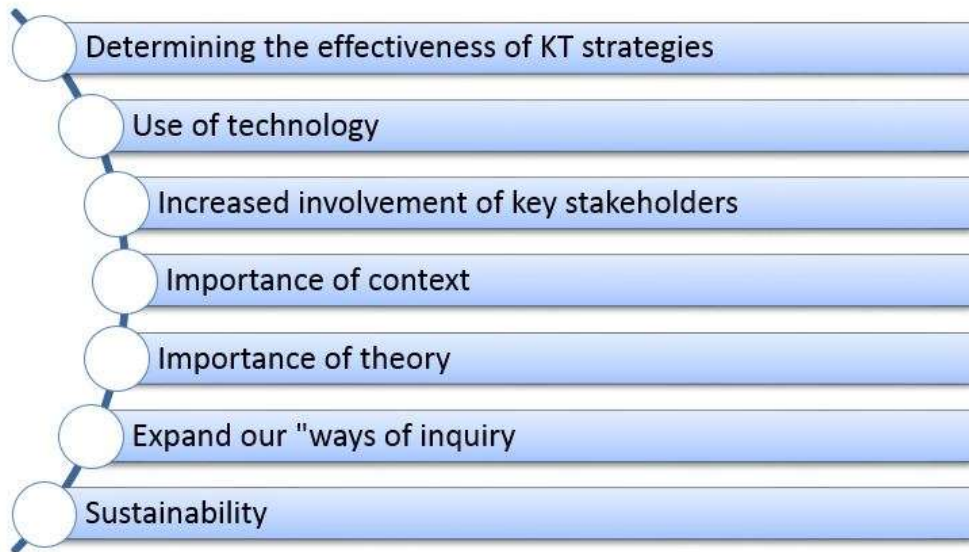

Supplement: Additional file 1: — Identified KT Priorities. Categorized seven main themes related to KT priorities [file 13012_2015_282_MOESM1_ESM.pdf]
